# Rapid highly sensitive general protein quantification through on-chip chemiluminescence

**DOI:** 10.1063/5.0039872

**Published:** 2021-04-29

**Authors:** Hoi Kei Chiu, Tadas Kartanas, Kadi L. Saar, Carina Mouritsen Luxhøj, Sean Devenish, Tuomas P. J. Knowles

**Affiliations:** 1 Department of Chemistry, University of Cambridge, Lensfield Road, Cambridge CB2 1EW, United Kingdom; 2 Fluidic Analytics Ltd., Unit A The Paddocks Business Centre, Cherry Hinton Road, Cambridge CB1 8DH, United Kingdom; 3 Cavendish Laboratory, Department of Physics, University of Cambridge, JJ Thomson Ave., Cambridge CB3 0HE, United Kingdom

## Abstract

Protein detection and quantification is a routinely performed procedure in research laboratories, predominantly executed either by spectroscopy-based measurements, such as NanoDrop, or by colorimetric assays. The detection limits of such assays, however, are limited to μM concentrations. To establish an approach that achieves general protein detection at an enhanced sensitivity and without necessitating the requirement for signal amplification steps or a multicomponent detection system, here, we established a chemiluminescence-based protein detection assay. Our assay specifically targeted primary amines in proteins, which permitted characterization of any protein sample and, moreover, its latent nature eliminated the requirement for washing steps providing a simple route to implementation. Additionally, the use of a chemiluminescence-based readout ensured that the assay could be operated in an excitation source-free manner, which did not only permit an enhanced sensitivity due to a reduced background signal but also allowed for the use of a very simple optical setup comprising only an objective and a detection element. Using this assay, we demonstrated quantitative protein detection over a concentration range of five orders of magnitude and down to a high sensitivity of $10\;\mathrm{pg}\;\mathrm{mL}{\;}^{-1}$, corresponding to pM concentrations. The capability of the platform presented here to achieve a high detection sensitivity without the requirement for a multistep operation or a multicomponent optical system sets the basis for a simple yet universal and sensitive protein detection strategy.

## I. INTRODUCTION

Proteins are central to a very wide range of biological processes,^1–3^ and, as such, their detection and quantification assays have become an integral part of biomolecular laboratory workflows. Some of the most widely used strategies in this context rely on recording the absorbance of protein samples in the UV wavelength range where the aromatic residues of proteins absorb and emit light. While serving as a fast and straightforward approach, the sensitivities of such assays have remained limited as the majority of the incident light is transmitted through the aqueous solution with only a very small fraction absorbed.^4^ More sensitive protein quantification can be achieved through the use of methods that yield colorimetric readouts. In this context, Commassie blue (Bradford) or bicinchoninic acid (BCA) assays, in particular, have become frequently employed methods in laboratory settings.^5,6^

The absorbance-based detection strategies that these assays use, however, pose inherent limitations on the lowest achievable detection sensitivities. This limit can be overcome and higher sensitivity reached with the use of fluorescence-mediated detection. In this context, both universal assays that allow generic protein detection^7^ and assays that rely on selectively detecting targets of interest via the inclusion of affinity reagents as can be achieved by enzyme-linked immunosorbent assays (ELISAs)^8^ are used. However, in both of these cases, factors inherent to fluorescence-based detection, such as fluctuations and noise from the excitation source,^9^ Raman and Rayleigh scattering events,^10^ and the limited photon budget of the sample^4^ present limitations to the achievable detection level and have led to the development of systems that involve multicomponent detection paths including optical filters or pinholes.

To overcome these limitations and develop a platform that combines highly sensitive protein detection and quantification with a simple setup, here, we devised and implemented a strategy that allowed proteins to be assayed through a chemiluminescent (CL) signal and implemented it on a microfluidic chip. Previously on-chip CL-detection assays have largely been immunoassay based (e.g., alphaLISA assays^11^) or chemically specific often involving the CL of luminol.^12–21^ This former strategy, in particular, has been applied in food analysis and point of care diagnostics.^22–25^ While such approaches afford very high sensitivities, they are designed to detect specific targets not permitting general purpose protein detection or quantification, and they are known to use reagents that are highly specific and expensive.^26^

Here, in contrast, we develop a platform for non-specific chemiluminescent protein detection and quantification not requiring specific reagents for each protein. Specifically, our presented platform relied on the generation of chemiluminescent through the use of naphthalene-2,3-dicarboxaldehyde (NDA) that activated proteins through their lysine residues. In addition to providing a route to generic protein detection, NDA is a fluorogenic molecule that ensured that the CL signal emerged latently and exclusively in the presence of proteins, rendering the assay free of manual washing steps. By custom-designing a microfluidic chip including a microfluidic mixer unit in which reactants could be mixed on timescales inaccessible by passive diffusion, we ensured that the assay can be performed by using minimal sample volumes. We validated the platform on assaying a variety of proteins and demonstrated quantitative detection over five orders of magnitude down to pM concentrations. In summary, our results provide a platform for highly sensitive generic protein detection in the format of a rapid single-step operation protocol and through the use of a simple detection platform comprising only detection systems but no excitation source.

## II. MATERIALS AND METHODS

### A. Protein samples

Bovine serum albumin (BSA), ovalbumin from egg white, β-lactoglobulin from bovine milk ( β-lac), lysozyme from chicken egg white, and the N α-Acetyl-l-lysine (NAK) were all purchased from Sigma-Aldrich. The calmodulin sample was kindly provided by Professor Sara Linse (Department of Biochemistry and Structural Biology, Lund University).

### B. Fabrication of microfluidic devices

**FIG. 1.**
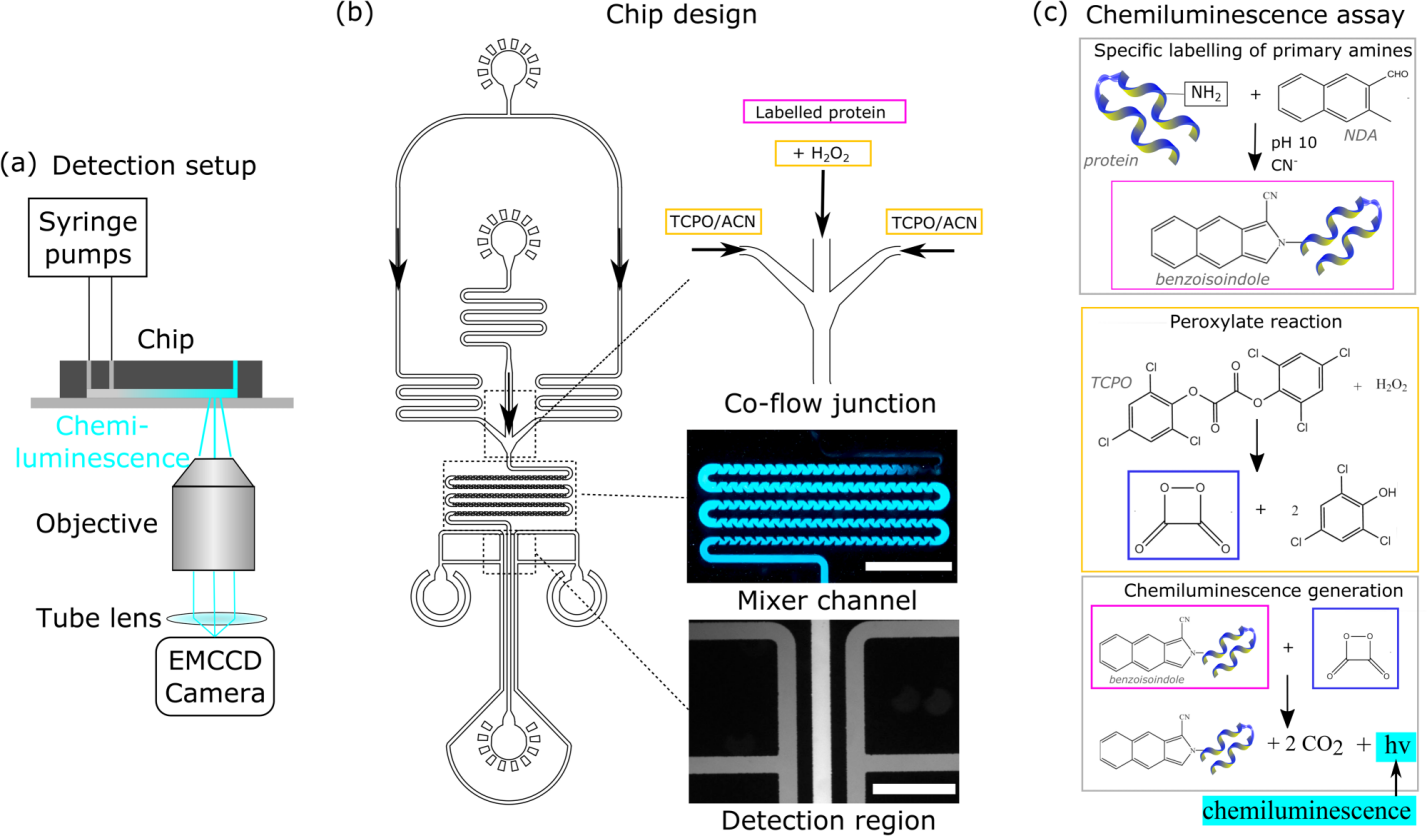
(a) The universal protein detection assay developed here relied on the generation of a chemiluminescent signal created in an excitation-free manner with the only required optical components being an objective and a detection element. (b) The assay was implemented on a microfluidic chip consisting of a co-flow junction (top inset), a mixer element comprising an array of cornered structures (middle inset; scale bar: 500μm), and a detection region (bottom inset; scale bar: 200μm). The mixing element was designed such that the two fluids would be fully mixed by the time they entered the detection region. The structures on both sides of the detection channel were filled with fluorescein to allow the imaging region to be precisely defined. (c) The chemiluminescent (CL) assay. Top box: Fluorogenic naphthalene-2,3-dicarboxaldehyde (NDA) reacted with the primary amines of proteins to form benzoisoindole (pink). Middle box: in parallel, a reaction between hydrogen peroxide and bis-(2,4,6-trichlorophenyl) oxalate (TCPO) yielded high-energy four-membered ring dioxetane (blue). Bottom box: When breaking down to CO ${}_{2}$, dioxetane transferred its energy to benzoisoindole, and the relaxation of benzoisoindole released a photon that was captured as a chemiluminescent (CL) signal. The fluorogenic nature of NDA rendered the assay free of a requirement to remove unbound probe molecules.

Microfluidic devices depicted in Fig. 1(b) were designed in AutoCAD and fabricated through conventional soft-lithography techniques.^27^ To this effect, SU-8 3050 photoresist (MicroChem) was spin-coated onto a silicon wafer to create a replica master with 50 μm high structures. The mold was then cast in polydimethylsiloxane (PDMS; Sylgard 184), and the patterned PDMS slab removed from the wafer and bonded to a glass slide using oxygen plasma treatment (Electronic Diener Femto; 15 s at 40% of maximum power). After the bonding process, the devices were exposed to an additional high-power plasma oxidation step^28^ for 500 s to render the channel walls more hydrophilic. The channels were then immediately filled with water, and their inlets and outlets blocked with gel-loading tips filled with water to prevent the recovery of the hydrophobic channel surfaces.

### C. Chemiluminescence (CL) measurements in microfluidic devices

First, a mixture consisting of 1.67 mM NDA and 1.67 mM KCN in 33.3 mM sodium borate buffer was prepared, and it was mixed in a 1:1 ratio with the protein sample to be analyzed to activate the protein through its lysine residues [Fig. 1(c), top box]. After a 2-min mixing time, the sample was dissolved in 32.6 M hydrogen peroxide ( $H_{2}O_{2}$) at the desired concentration. To initiate the CL reaction, the labeled protein sample was injected to the microfluidic chip from one of its inlets [Fig. 1(b)] and bis-(2,4,6-trichlorophenyl) oxalate (TCPO), dissolved in acetonitrile (ACN) to its solubility limit, injected from the other inlet. A flow rate of $3\;\mathrm{mL}\;h^{-1}$ was used for both fluids. The solutions were loaded using 500μL glass syringes (Hamilton UK) and connected to the microfluidic chip through 27 gauge needles (Neolus Terumo, UK) and polyethene tubing (Scientific Laboratory Supplies, UK; inner diameter of 0.38 mm and outer diameter of 1.09 mm). The fluids were injected into the devices using a syringe pump (Nemesys, Cetoni GmbH). After meeting at the T-junction, the solutions entered a micromixer unit [Fig. 1(b), middle inset]. Finally, fluorescein solution was injected into the two side channels of the device positioned parallel to the measurement area [Fig. 1(b), bottom inset). This enabled the measurement area to be precisely located.

### D. Flow simulations

To model the behavior of the fluids in the microfluidic devices, the micromixer unit was simulated using a COMSOL Multiphysics 5.2a finite element method (FEM) analysis. The simulations were based on three constraints: (i) the continuity equation, (ii) the Navier–Stokes equation, and (iii) the convection–diffusion equation as described in more detail in the supplementary material. The solution to the equations was obtained in two steps: first, the fluid flow field in the channel was computed ( $7.7\times 10^{6}$ degrees of freedom), and this flow field was subsequently used to compute the analyte convection and diffusion along the channel ( $6.4\times 10^{6}$ degrees of freedom). For simplicity, single phase flow simulation was implemented with fluid properties similar to aqueous buffers: density of ρ=1000 kg m ${}^{-3}$ and dynamic viscosity of $\mu =10^{-3}$ Pa s.

### E. Data acquisition and analysis

First, a fluorescence image of the region enclosed by the side channels was acquired [Fig. 1(b), bottom inset]—this micrograph served as a reference to define the imaging position. Next, the filter cubes were removed from the system, and an image of the same region was recorded without using an illumination source. This image was recorded using a 70 s long exposure time (Photometrics Evolve 512) coupled to a 10 × objective, and it defined the background luminescence level of the measurement, $I_{0}$. Finally, the reagents were introduced to the chip as described in Sec. II C, and the chemiluminescence from the protein recorded similarly over a 70-s long exposure time. This image defined the chemiluminescence generated by the analyzed protein sample, $I_{\;\mathrm{protein}}$. The intensities of the latter two measurement points were then subtracted from each other, $I=I_{\;\mathrm{protein}}-I_{0}$, to yield a background corrected readout.

## III. RESULTS AND DISCUSSION

### A. Chemiluminescent assay

To develop a strategy for non-specific protein detection, we exploited on the general presence of lysine groups in proteins. This characteristic is employed as part of fluorescent labeling strategies both when conventional multi-step labeling strategies, such as Alexa-dye based labeling, are used as well as in single-step assays that use fluorogenic dyes where fluorescent signals emerge exclusively in the presence of proteins, which sets the basis for single-step purification-free operation.^7^ While permitting quantitative protein detection, fluorescence-based protein assays have experienced limits in their sensitivities due to high levels of background. This effect is particularly pronounced when the measurements are performed on chip because the majority of the polymers used for replicable and scalable production of micron scale devices are known to exhibit some degree of autofluorescence.^29,30^

To overcome this limitation, we similarly used a fluorogenic dye, NDA, that formed a fluorescent conjugate with the protein, benzoisoindole [Fig. 1(c), top box]. Instead of directly detecting the fluorescence, we allowed benzoisoindole to react with a high-energy intermediate, dioxetane, formed in a reaction between TCPO and H ${}_{2}$O ${}_{2}$ (middle box). When the highly unstable dioxetane broke down, it excited benzoisoindole that in turn emitted a chemiluminescent signal when returning to its ground state [Fig. 1(c), bottom box]. Crucially, the fluorogenic nature of NDA ensured that the signal emerged exclusively in the presence of the protein, which rendered the assay free of washing steps.

### B. Microfluidic device design

To execute this chemiluminescent signal generation and detection strategy on a microfluidic chip, we first investigated the performance of the system by co-flowing an NDA-labeled protein sample mixed with hydrogen peroxide and a mixture of bis-(2,4,6-trichlorophenyl) oxalate (TCPO) and acetonitrile (ACN) into a chip that included just the T-junction [Fig. 1(b), top inset] but no mixing unit. While we observed the emergence of chemiluminescence as expected, the signal was visible only at the center of the channel where the two streams met even when very low injection flow rates were used. Indeed, for this simple geometry, mass transport between the two fluids occurred only by diffusive means, and this effect was insufficient to provide a complete mixing between the streams within their limited residence time in the device. Furthermore, such inefficient mixing does not only waste a large proportion of the reagents. More importantly, as only some fraction of the protein present in the sample participates in the reaction, the number of photons that is generated does not reach its maximum value, which strongly affects the achievable sensitivity limit of the system and prohibits us from developing a quantitative protein detection assay.

To overcome this limitation, we explored various on-chip mixing strategies. First, attempts to induce active mixing were taken by incorporating a 0.5 mm magnetic stirrer bar to the chip. While this strategy proved to be an effective means to introduce mixing, the positioning of both the stirrer bar and the magnet was too delicate for it to act as a reliable strategy for robust on-chip measurements.

**FIG. 2.**
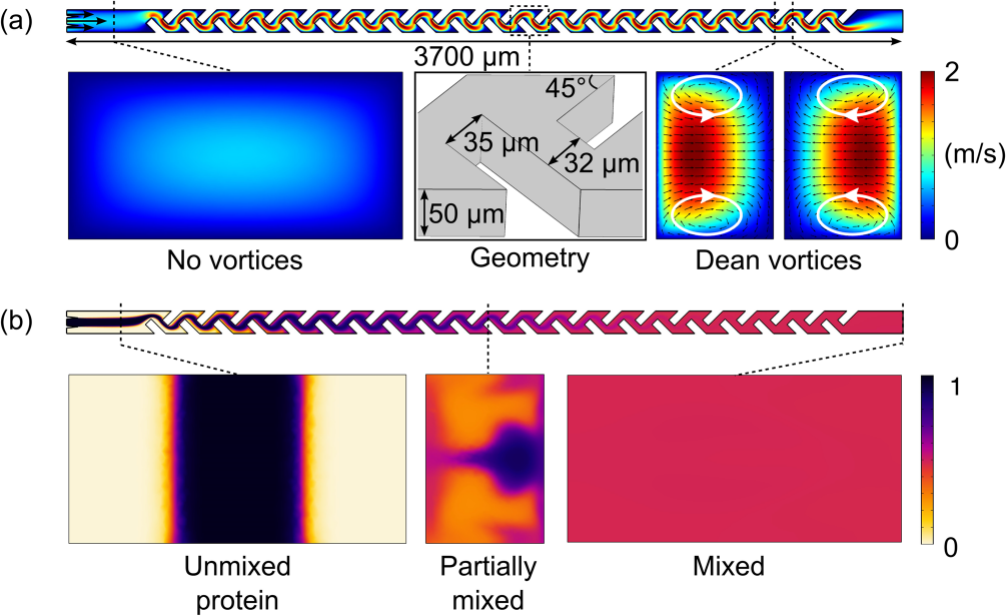
Simulated performance of the micromixer unit at $Q_{\mathrm{mix}}=6000\;\mu L\;h^{-1}$, the flow rate used in this study. (a) When the fluid first entered the micromixer unit through a straight channel, no vortices were predicted to occur across the cross section of the channel (left inset). When the fluid was forced to change the flow direction around periodic sharp edges at a high velocity (middle inset), Dean vortices were created (right inset), which permitted effective mixing of the two fluids. (b) Variations in the protein concentration across the micromixer unit. Initially, an unmixed protein was surrounded by the reactant streams from two sides. The protein was gradually mixed with the rest of the fluid when the fluids moved through the micromixer. By the time the fluids reached the end of the mixing channel, the protein was predicted to be uniformly spread across the device for the conditions and the device geometry used here.

We thus set out to integrate with the microfludic device design a dedicated mixing unit that would allow achieving more efficient and faster mixing than was possible by means of diffusion. Specifically, we decided to use an approach where the fluids to be mixed were moved around sharp corners where they were forced to change the direction of flow [Fig. 2(a)] as has been previously described by Arnon *et al.*^31^ In its original implementation, this device geometry had been used at a slow flow rate, approximately $2\;\mu L\;h^{-1}$. When we attempted to implement the chemiluminescent protein detection assay under these conditions, we observed protein precipitation within the device, which eventually blocked the microfluidic channels. To find conditions at which this device geometry would allow effective mixing also at higher flow rates, we modeled the behavior of the fluids in the device using CFD simulations (see Sec. III C). In contrast to the active mixing approach examined earlier, such an on-chip mixing strategy provided the advantage of not requiring the inclusion of any external components, thereby ensuring that the devices can be fabricated through a single-step lithography process in a highly reproducible manner.

### C. Flow simulations

While a strategy that mixes fluids by forcing them to move through periods of sharp edges [Fig. 1(b)] has been used before,^31^ no guidance for choosing optimum device geometry and flow rates has been described to the best of our knowledge. Thus, to find suitable operating conditions and ensure efficient use of the chemical reagents and the protein sample, we performed computational fluid dynamics (CFD) simulations using COMSOL Multiphysics 5.2a as described in Sec. II. Specifically, we examined the behavior of the mixing unit by modeling the movement of a molecule with a diffusion coefficient of $D=10^{-11}\;m^{2}\;s^{-1}$, corresponding to a hydrodynamic radius of $R_{h}=21\;\mathrm{nm}$. For proteins of a smaller hydrodynamic radius and hence a higher diffusion coefficient value, the extent of mixing would be larger. Hence, using $D=10^{-11}\;m^{2}\;s^{-1}$ as the diffusion coefficient allowed us to design a device that provided effective mixing for all molecules up to 21 nm—a range that covers the majority of proteins.

The results of our simulations at a fixed flow rate of $Q_{\mathrm{mix}}=6000\;\mu L\;h^{-1}$ illustrated that in the straight section of the channel that no vortices occurred [Fig. 2(a), left inset] and the fluids remained unmixed [Fig. 2(b), left inset]. However, when the fluid was forced to change the flow direction around the periodic sharp edges of the micromixer unit [Fig. 2(a), middle inset], vortices were created [Fig. 2(a), right inset), which in turn induced mixing between the two streams [Fig. 2(b), middle and right insets].

These trends illustrate that while larger flow rates led to a reduced residence time in the device, they also resulted in stronger vortices. To understand the balance between these effects and evaluate how the efficiency of mixing varies with the flow rate, we simulated the mixing process under a variety of flow rates from 4 to 6000 μL h ${}^{-}1$. For the geometry of our micromixer unit [dimensions illustrated in Fig. 2(a)], these flow rates corresponded to Reynolds numbers ( Re) from 0.016 to 47. To describe the efficiency of the mixing process, we evaluated the mixing index (MI) for all the simulated conditions using the following equation:^32^

$$MI=\left[1-\sqrt{\frac{\int \int_{A}{\left(c-c_{\mathrm{mix}}\right)}^{2}\cdot dA}{\int \int_{A_{0}}{\left(c_{0}-c_{mix}\right)}^{2}\cdot dA}}\;\;\right]\times 100\text{\%},$$ (1)

where $c_{0}$ is the original unmixed concentration at the inlet cross section $A_{0}$, c is the species concentration distribution on the selected cross section A, and $c_{mix}$ is the concentration of the solutions to be mixed, which was set to $c_{\mathrm{mix}}=5\;\mu M$ in this simulation. For such a system, mixing index MI=0% represents no mixing, while a mixing index of MI=100% corresponds to full mixing.

**FIG. 3.**
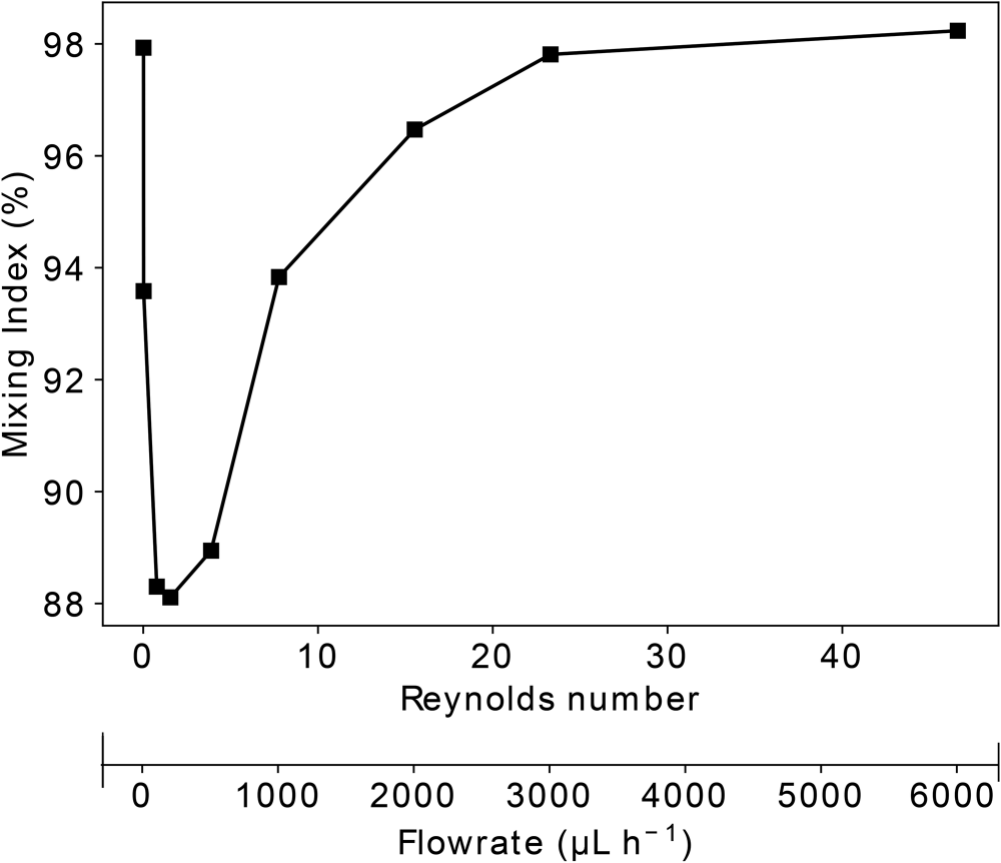
Simulated mixing index (MI) at the outlet of the micromixer unit as a function of Reynolds number, Re, and the total flow rate of the fluids in the device. Based on these simulation results, a total flow rate of $Q_{\mathrm{mix}}=6000\;\mu L\;h^{-1}$ was used ( Re=47). An elevated flow rate would have not resulted in enhanced mixing, but it would have led to a larger reactant consumption and a larger pressure drop across the device.

From the estimated mixing indices (Fig. 3), we concluded that at low Reynolds numbers ( <1), the mixing index decreased with increasing flow rate, indicating that the process was dominated by diffusional movement where a decreased residence time of the fluid in the micromixer unit led to a lower degree of mixing. At higher Reynolds numbers, however, the trend in the decreasing mixing index with increasing flow was reversed. This result can be explained by inertial effects gaining importance over diffusive ones. Exploiting this effect allowed us to achieve good mixing between the two streams even though their residence times in the device were small.

Based on these simulation results, we set out to use a combined flow rate of $Q_{\mathrm{mix}}=6000\;\mu L\;h^{-1}$ for the micromixer unit. A further increase in the flow rate would not result in a substantially enhanced mixing (Fig. 3) but would increase the total pressure drop in the system. Under these conditions, the two streams of fluids can be expected to be nearly completely mixed ( MI=98%) by the end of the first stretch of the mixing channel. We note that the mixing behavior simulations served as an approximation of the exact device operation as they were performed by modeling the fluids as a single phase. However, our experimental results [Fig. 1(b), middle inset], where we observed a chemiluminescence signal already within the first five turns of the mixing unit, indicated that the approximation to treat the fluids as a single phase was a valid simplification for obtaining first-order estimates.

### D. Chemiluminescent protein detection and quantification on chip

Having established the micromixer unit, we examined the possibility to use our developed platform for protein detection and quantification. In particular, for the system to serve as an effective analytical platform, it is essential that the CL intensity emitted from the sample can be related to its concentration.

**FIG. 4.**
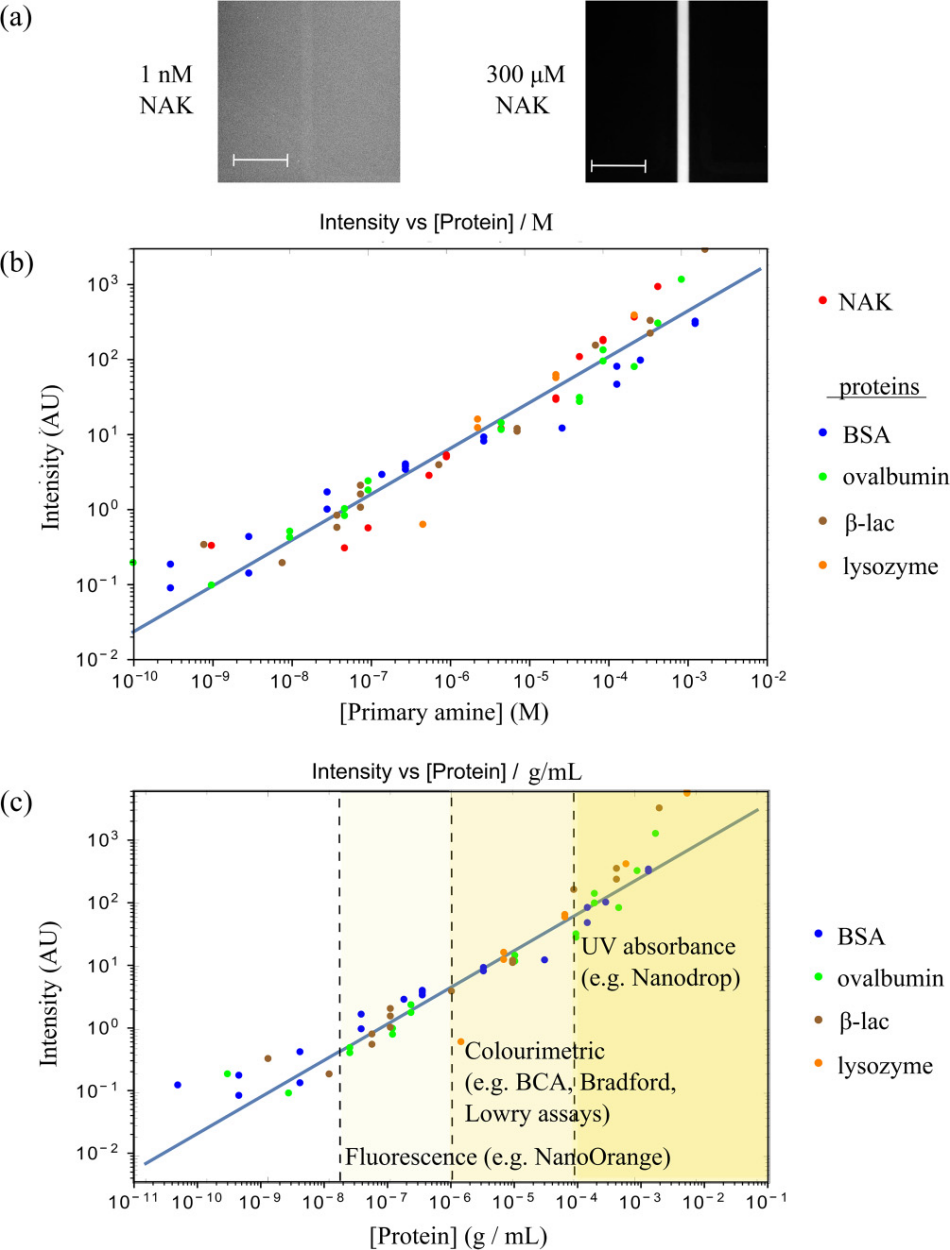
Quantification of protein concentration. (a) CL images of N α-acetyl-l-lysine (NAK) at the lowest (left) and highest (right) concentration studied; scale bar is 200μm. (b) CL signal as a function of the primary amine concentration. The red points represent the measurements performed on N α-Acetyl-l-lysine (NAK) and the remaining four colors to proteins of various physicochemical properties. The CL intensity varied linearly with primary amine concentration over five orders of magnitude, with the lowest detected value at 300 pM primary amines, which translated to 5 pM BSA. The blue line is a best fit linear relationship over five sets of data. (c) In addition to showing linear scaling with the concentration of primary amines, CL intensity was further seen to scale with the mass concentration of the proteins. These data demonstrate the capability of the platform to serve as a general strategy for measuring protein concentrations with its sensitivity limit exceeding what can be reached by conventional protein quantification assays relying on fluorescence,^5^ colorimetric signals,^6^ or absorbance^33^ as indicated by the vertical dotted lines, respectively.

With the labeling reaction occurring through the amine groups of lysine residues, we first set out to examine the concentration dependent behavior of N α-acetyl-l-lysine (NAK) in our device, as it included only a single amino acid with a primary amine. To this effect, we performed experiments at a range of NAK concentrations between 1 nM and 300 μM and plotted the emitted CL signal for each case. From these data, we observed the CL intensity to vary linearly with the primary amine concentration [Fig. 4(b), red circles], setting the basis for the hypothesis that the system has could serve as a quantitative protein detection system. Using the lowest concentration at which NAK was analyzed, 1 nM [Fig. 4(a), left image] and extracting the average signal across the image for the background ( 88.6±33) and for the detection region ( 139.8±28), we estimated the signal from the sample to be 51.2±43.5 AUs (errors were added in quadrature), which provides an estimate for the uncertainty in the measurement of around 0.3 AU $\cdot \frac{43.5}{51.2}=0.25$ AU at this lowest concentration [Fig. 4(b), red circles].

Next, a range of proteins (bovine serum albumin, ovalbumin, β-lactoglobulin, and lysozyme) and their chemiluminescent responses on the microfluidic platform were investigated. For all the four proteins, the CL signals were recorded at a number of concentrations, and the observed intensities were similarly plotted as a function of the primary amine concentration [Fig. 4(b)]. We observed that the detected signal varied linearly with the protein concentration as desired. The lowest detected concentration was around 300 pM of primary amines or around 5 pM BSA [Fig. 4(b), blue circles].

We further observed from Fig. 4(b) that the recorded CL intensity did not only scale with the protein concentration, but also gave a direct readout for the concentration of primary amines in the sample. This feature suggested that the platform had a further potential to determine absolute concentrations of protein samples. We tested this hypothesis by assaying calmodulin samples of two different concentrations. Calmodulin is a protein involved in signaling pathways and a key regulator of the concentration of calcium ions. Its levels thereby control a variety of important biological processes ranging from muscle contraction^34^ and axonal flow to endo- and exocytosis.^35^ By injecting a 15 μM (0.25 mg mL ${}^{-1}$) calmodulin sample to our designed system, we measured a CL intensity of 107.9±4.0 a.u., translating to ( 0.26±0.017) mg mL ${}^{-1}$ of protein ( n=3 measurements were performed using three different microfluidic chips; error refers to standard deviation). Another experiment was repeated for a 1.5μM calmodulin (0.025 mg mL ${}^{-1}$ protein) sample. A CL intensity of 26.9 ± 2.7 a.u. was recorded, translating to ( 0.022±0.004) mg mL ${}^{-1}$ of protein, which similarly was in good agreement with the expected concentration.

As can be seen from Fig. 4(c), our described microfluidic chemiluminescent protein detection and quantification strategy is capable of determining protein concentrations over five orders of magnitude and down to $10\;\mathrm{pg}\;mL^{-1}$ concentration. To the best of our knowledge, this detection limit is a marked improvement over established methods for measuring protein concentrations, such as Pierce 660 assay, Bradford assay, absorbance-based detection on the Nanodrop platform, a Lowry assay, and a bicinchoninic acid assay, which have their lower limits for detection at 50 μ g mL ${}^{-1}$, 15 μg mL ${}^{-1}$, 10 μg mL ${}^{-1}$, 1 μg mL ${}^{-1}$, and 0.5 μg  mL ${}^{-1}$, respectively.^5,6,33,36^ Moreover, $10\;\mathrm{pg}\;{\mathrm{mL}}^{-1}$ as currently demonstrated is not a hard limit for such a general CL-based protein detection strategy as the lowest limit of detection was determined by the objective and the sensitivity of the camera. We predict that lower concentrations of proteins can be probed with a more sensitive CCD camera or a PMT.

Finally, we note that while the experiments performed here involved off-chip labeling of the protein sample with the NDA dye, this reaction is relatively fast ( <2 min) and does not require a purification step and could, therefore, also be performed directly on the chip. As such, through the inclusion of an additional inlet next to the sample injection port, it would be possible to inject unlabeled samples.

## IV. CONCLUSIONS

We demonstrated a strategy for general and highly sensitive detection of proteins that was quantitative over five orders of magnitude and down to concentrations of 10 $\mathrm{pg}\;{\mathrm{mL}}^{-1}$—the result was a marked improvement over incumbent universal protein detection assays that have their detection limits in the $100\;\mu \;g\;{\mathrm{mL}}^{-1}$ range for UV-absorbance and the $1\;\mu g\;{\mathrm{mL}}^{-1}$ range for colorimetric assays. We achieved this objective by establishing a chemiluminescent assay that detected proteins through their lysine residues and implemented it on a microfluidic platform that through the inclusion of a micromixer unit ensured effective mixing of the reactants beyond which it would be possible by means of simple diffusion. In contrast to conventional fluorescence-mediated detection approaches, our demonstrated platform operated without the inclusion of an excitation source, which greatly simplified the optical setup, reducing it down to only a detection component. Moreover, the latent nature of the assay ensured that the platform can be operated without the need for a washing step. Due to the general presence of amine groups in proteins, the sensing strategy demonstrated here is universally applicable to any protein sample.

## SUPPLEMENTARY MATERIAL

See the supplementary material for details about the computational fluid dynamics simulations.

## Data Availability

The data that support the findings of this study are available within the article. Raw micrographs are available from the corresponding author upon reasonable request.
